# Bridging the gap between clear cell renal cell carcinoma and cutaneous melanoma: the role of SCARB1 in dysregulated cholesterol metabolism

**DOI:** 10.18632/aging.205083

**Published:** 2023-10-05

**Authors:** Lebin Song, Shuai Wang, Xi Zhang, Ninghong Song, Yan Lu, Chao Qin

**Affiliations:** 1 Department of Dermatology, The First Affiliated Hospital of Nanjing Medical University, Nanjing 210029, China; 2 Department of Urology, The State Key Lab of Reproductive, The First Affiliated Hospital of Nanjing Medical University, Nanjing 210029, China

**Keywords:** cholesterol metabolism, clear cell renal cell carcinoma, prognostic model, SCARB1, skin cutaneous melanoma, tumor immune

## Abstract

Objective: The metabolism of cholesterol has been found to be closely related to the proliferation, invasion, and metastasis of tumors. The purpose of this study was to investigate the correlation between cholesterol metabolic genes and the prognosis of clear cell renal cell carcinoma (ccRCC).

Methods: Gene expression profiles and clinical information of individuals diagnosed with prevalent malignant tumors were obtained from the TCGA database. For survival analysis, Kaplan-Meier curves were used. Consensus clustering was utilized to identify distinct molecular clusters. LASSO regression analysis was utilized to construct a novel prognostic signature. Differential analysis was used to analyze the differences in gene expression and various evaluation indicators between different subgroups. RT-qPCR and Immunohistochemistry were performed to examine the gene expression. Small interfering RNA transfection, CCK-8, and clone formation assays were conducted to verify the function of the target gene in ccRCC cell lines.

Results: Based on genes involved in cholesterol metabolism related to survival, two molecular ccRCC subtypes were identified with distinct clinical, immune, and biological features. A molecular signature which would be utilized to evaluate the prognosis and the immune status of the tumor microenvironment of ccRCC patients was also established. The SCARB1-mediated cholesterol-dependent metabolism occurred both in ccRCC and skin cutaneous melanoma.

Conclusion: A gene signature related to cholesterol metabolism was developed and validated to forecast the prognosis of ccRCC, demonstrating a correlation with immune infiltration. Cholesterol metabolic genes such as SCARB1, were expected to contribute to the diagnosis and precision treatment of both ccRCC and skin cutaneous melanoma.

## INTRODUCTION

Cholesterol plays a vital role in the structure of eukaryotic cell membranes and governs the physiological characteristics of these membranes. It has been demonstrated that disorders of cholesterol metabolism contribute to many diseases including diabetes, cardiovascular disease, and cancers [1–4]. Cholesterol homeostasis in the body is primarily regulated by the biosynthesis of endogenous cholesterol and the uptake of exogenous cholesterol. Studies have shown that elevated levels of cholesterol in the blood were associated with an increased likelihood of developing various types of cancer, including colon, rectal, prostate, and testicular cancers [3, 5]. Expression of multiple genes involved in the synthesis and uptake of cholesterol, such as 3-hydroxy-3-methylglutaryl coenzyme A reductase (HMGCR), is significantly upregulated in tumor tissues [6–8].

Kidney cancer, characterized by a rising incidence and onset at a younger age is a prevalent form of malignancy in the urinary system among adults, resulting in approximately 403,000 fresh instances and 175,000 fatalities globally during 2018 [9, 10]. Clear cell renal cell carcinoma (ccRCC), accounting for 70–80% of all types of kidney cancer, shows varying prognosis among patients at different stages. Early-stage ccRCC patients typically have a favorable outlook when treated surgically, while individuals with advanced, metastatic, or recurrent ccRCC often face an unfavorable prognosis due to the lack of effective treatment options [11, 12]. ccRCC is distinguished by the presence of abundant intracellular lipid droplets (LDs) that contain high levels of both free and esterified cholesterol [13, 14]. It is conceivable that the resistance of ccRCC to treatment partly arises from the accumulation of cholesterol. However, to date, there has been a paucity of relevant studies concerning the predictive significance of the genetic patterns associated with cholesterol balance in ccRCC.

With the rapid advancement of molecular biology and sequencing technology, there has been a recent discovery of certain single-gene biomarkers that can be used to predict the prognosis of renal cell carcinoma (RCC) [15–17]. However, the expression of these individual genes is susceptible to various influences, potentially limiting their predictive performance. Therefore, examining numerous genes controlled by the identical crucial pathway in order to establish a proficient gene signature could potentially enhance predictive accuracy.

In the current study, we identified novel cholesterol metabolic subtypes and constructed a highly stable cholesterol metabolic prognostic signature in ccRCC, representing various immune characteristics in the tumor microenvironment. In addition, we also found the exogenous cholesterol dependence of ccRCC, which count on SCARB1 mediated high-density lipoprotein (HDL) intake, also existed in skin cutaneous melanoma (SKCM). Therefore, this metabolic fragility and its mediator SCARB1 are expected to be the diagnostic biomarker and therapeutic target of ccRCC and SKCM.

## METHODS

### Data acquisition

The RNA-sequencing data and corresponding clinical information of 539 ccRCC and 72 normal tissue samples were obtained from The Cancer Genome Atlas (TCGA) website (https://www.cancer.gov/tcga). Matrix files of gene expression profiles and clinical information of the E-MTAB-1980 cohort, including 101 ccRCC tissue samples, were obtained from the ArrayExpress website (https://www.ebi.ac.uk/arrayexpress). A list of 10 genes encoding proteins participating in cholesterol metabolic pathways was summarized from the published article [18]. We defined these genes as cholesterol metabolic genes (CMGs) in this article.

### Patients and specimens

30 ccRCC specimens and paired non-tumor adjacent tissue specimens were collected from patients who underwent surgery at The Affiliated Hospital of Nanjing Medical University, Nanjing, China between December 2022, and February 2023. All the specimens were confirmed independently by two experienced pathologists. All specimens were freshly snap-frozen in liquid nitrogen and stored in a deep freezer at −80°C for RNA and protein extraction. All patients met the criterion of not receiving chemotherapy before the surgery. Each patient signed an informed consent form prior to participation. Paraffin-embedded tissues were provided by the Department of Pathology of the hospital. This study was approved by the Ethics Committee of The Affiliated Hospital of Nanjing Medical University.

### Differential analysis

Regarding |log2 fold changes (FC)|≥1 and adjusted *P*-value < 0.001 as the specific criteria, we utilized the R package limma to screen out the differentially expressed genes (DEGs) between two groups.

### Cluster analysis

The molecular subtypes of ccRCC samples were identified by the consistent clustering method [19]. The clustering algorithm is set to K-means and the coefficients *k* are set from 2 to 9. The optimal clustering result was presented as a heat map including the expression of clustering genes and the distribution of classical clinical parameters across cases.

### Survival analysis

Clinical outcomes were assessed by calculating the differences using the Log-rank test via the Kaplan-Meier (KM) method. Prognostic analysis was conducted using various R packages, such as KMsurv, survival, and survminer. A *P*-value less than 0.05 was deemed to be statistically significant.

### Enrichment analysis of biological pathway and function

DEGs between two clusters were screened out through the differential analysis. To estimate the variation in pathways and biological process activities between different cholesterol subtypes, Gene Ontology (GO) and Kyoto Encyclopedia of Genes and Genomes (KEGG) enrichment analyses were performed based on the GO database (http://geneontology.org) and KEGG database (http://www.genome.jp/kegg/) in R package clusterProfiler. Gene Set Variation Analysis (GSVA) was performed to evaluate the pathways enriched in each cluster with the R package *GSVA* [20] and “c2.cp.kegg.v7.4.symbols” from the Molecular Signatures Database (MSigDB). Significance was defined according to the nominal *P*-value < 0.05 and false discovery rate (FDR) <0.05.

### Analysis of immune infiltration and function

Eight algorithms were used to compare the immune infiltration status between subgroups, including the ssGSEA algorithm and the corresponding gene sets used [21]. The ESTIMATE algorithm allowed for quantitative comparison of immune correlation status among GMRS subgroups using 4 kind scores [22].

### Construction of CMGs-based cholesterol metabolic signature

The univariate Cox regression analysis was conducted to find out prognosis-related candidate CMGs, with a *P*-value < 0.05 considered statistically significant. Then, the least absolute shrinkage and selection operator (LASSO) Cox regression analysis was conducted to shrink the scope of gene screening, identify highly correlated CMGs, and construct a prognostic gene signature with R package glmnet. Using the linear combination of gene expression weighted regression coefficients, we got the risk score formula for the prognostic model of CMGs.

### Evaluation of CMGs-based cholesterol metabolic signature

The ccRCC patients with survival data were divided into a high-risk group and a low-risk group based on the median value of the risk score. The KM survival curve and time-dependent receiver operating characteristic (ROC) curve were plotted to evaluate the prediction efficiency of CMG signature, which was drawn by the survival and timeROC R packages, respectively. Hereafter, univariate and multivariate Cox regression analyses were used to evaluate the independence of prognostic gene signature and other clinical parameters. Correlation and survival analyses in the stratification of different clinical features were performed to validate the prediction capability of our model. In addition, we also explored the correlation of our risk score with immune cell infiltration and common immune checkpoints.

### Cell culture and knockdown of target genes

Human ccRCC cell lines 786-O, 789-P, Caki-1, and the human proximal tubule epithelial cells HK-2 were obtained from the University of Colorado Cancer Center Cell Bank. Human ccRCC cell lines were cultured in RPMI-1640 medium supplemented with 10% fetal bovine serum (FBS, Invitrogen, Carlsbad, CA, USA) at 37°C in a 5% CO2 atmosphere. The HK2 cell line was grown in Keratinocyte Serum Free Medium (Thermo Fisher Scientific, Waltham, MA, USA) supplemented with 0.05 mg/mL bovine pituitary extract and 5 ng/mL epidermal growth factor. To knock down the expression of target genes, Lipofectamine RNAiMAX transfection reagent and Opti-MEM (Thermo Fisher Scientific) were used to transfect the small interfering RNAs (siRNAs) into ccRCC cell lines. The sequences of si-SCARB1 were listed in Table 1.

**Table 1:** Primer sequences of associated genes for reverse transcription-quantitative polymerase chain reaction.

**Gene**	**Sequence**
SCARB1	Forward: 5′-CCTATCCCCTTCTATCTCTCCG-3′
	Reverse: 5′-GGATGTTGGGCATGACGATGT-3′
GAPDH	Forward: 5′-GGTGAAGGTCGGAGTCAACGG-3′
	Reverse: 5′-GAGGTCAATGAAGGGGTCATTG-3′
si-SCARB1	Sense: 5′-CAAGUUCGGAUUAUUUGCUTT-3′
	Antisense: 5′-AGCAAAUAAUCCGAACUUGTT-3′

### RNA extraction and real-time quantitative reverse transcription PCR

Total RNA from cells and tissues was isolated with TRIzol Reagent (Invitrogen, Carlsbad, CA, USA) based on manufacturers. cDNA was obtained from total RNA with PrimeScript™ RT reagent kit (Takara Bio, Inc., Otsu, Japan). The mRNA expression was assessed by Real-time quantitative PCR, which was carried out in triplicate by an SYBR Premix Ex Taq™ kit (Takara Bio) and ABI 7900HT Real-Time PCR system (Applied Biosystems Life Technologies, Foster City, CA, USA). The amplification reaction conditions for SCARB1 and glyceraldehyde-3-phosphate dehydrogenase (GAPDH) were 10 min at 95°C, 45 cycles at 95°C for 10 s, then 10 s at 60°C and 15 s at 72°C. The GAPDH gene served as an endogenous control. The relative expression of SCARB1 was analyzed by normalizing it to internal control GAPDH using the 2^−ΔΔCt^ method. The primer sets for SCARB1 and GAPDH were listed in Table 1.

### Cell proliferation and clone formation

For the cell proliferation assay, 3 × 10^3^ cells suspended in 100 ul RPMI-1640 medium were seeded into 96-well plates. The cell proliferation was assessed by the CCK8 (Dojindo Molecular Technologies, Japan). 10 μl CCK8 solution was given to each well plate after different incubation times: 0 hours, 24 hours, 48 hours, and 72 hours. Finally, we measured the absorbance at 450 nm wavelength after 2 hours of incubation. For the clone formation assay, 500 cells were seeded into 6-well plates and incubated at 37°C. Clone size was observed daily under a microscope until the number of cells in the majority of clones was >50. Then, the medium was removed, and the cells were stained with 0.2% crystal violet for 30 min. The cells were washed 3 times with PBS, then photographed and the clones were counted.

### Immunohistochemistry (IHC) staining

IHC was performed on paraffin-embedded sections according to standard protocols. Briefly, tissues were fixed in 4% paraformaldehyde solution or 10% neutral-buffered formalin at room temperature overnight and paraffin-embedded following standard procedures. The sections were deparaffinized in xylene and hydrated with decreasing concentrations of ethanol (100, 90, 80, 75%) for 3 min each time and microwaved-heated in sodium citrate buffer for antigen retrieval. Then, the sections were blocked in 5% BSA and incubated with primary antibodies: anti-LDHA (1:100 dilution, Cell Signaling Technology, Inc., Danvers, MA, USA #3582), anti-CD33 (1:100 dilution, ProteinTech Group, Inc., Wuhan, China, #17425), anti-LOX-1 (1:200 dilution, ProteinTech Group, Inc., #11837), or anti-c-Rel (1:100 dilution, Santa Cruz Biotechnology, USA, #sc-6955), followed by incubation with horseradish peroxidase-conjugated goat anti-rabbit secondary antibody. Antibody binding was visualized using a 2-Solution DAB Kit (Invitrogen). The images were obtained with an inverted microscope (Olympus IX71, Japan). An H-score was calculated using the following formula: H-score = ∑ (PI × I) = (percentage of cells of weak intensity × 1) + (percentage of cells of moderate intensity × 2) + percentage of cells of strong intensity × 3). Here H-score was recorded as a continuous variable.

### Statistical analysis

All analyses were performed using R software (version 4.1.2). All statistical tests were two-sided. *P*-value < 0.05 or Spearman correlation coefficient > 0.3 was considered statistically significant unless otherwise noted.

## RESULTS

### Identification of cholesterol metabolic subtypes and their correlation with biological functions in ccRCC

**Figure 1:**
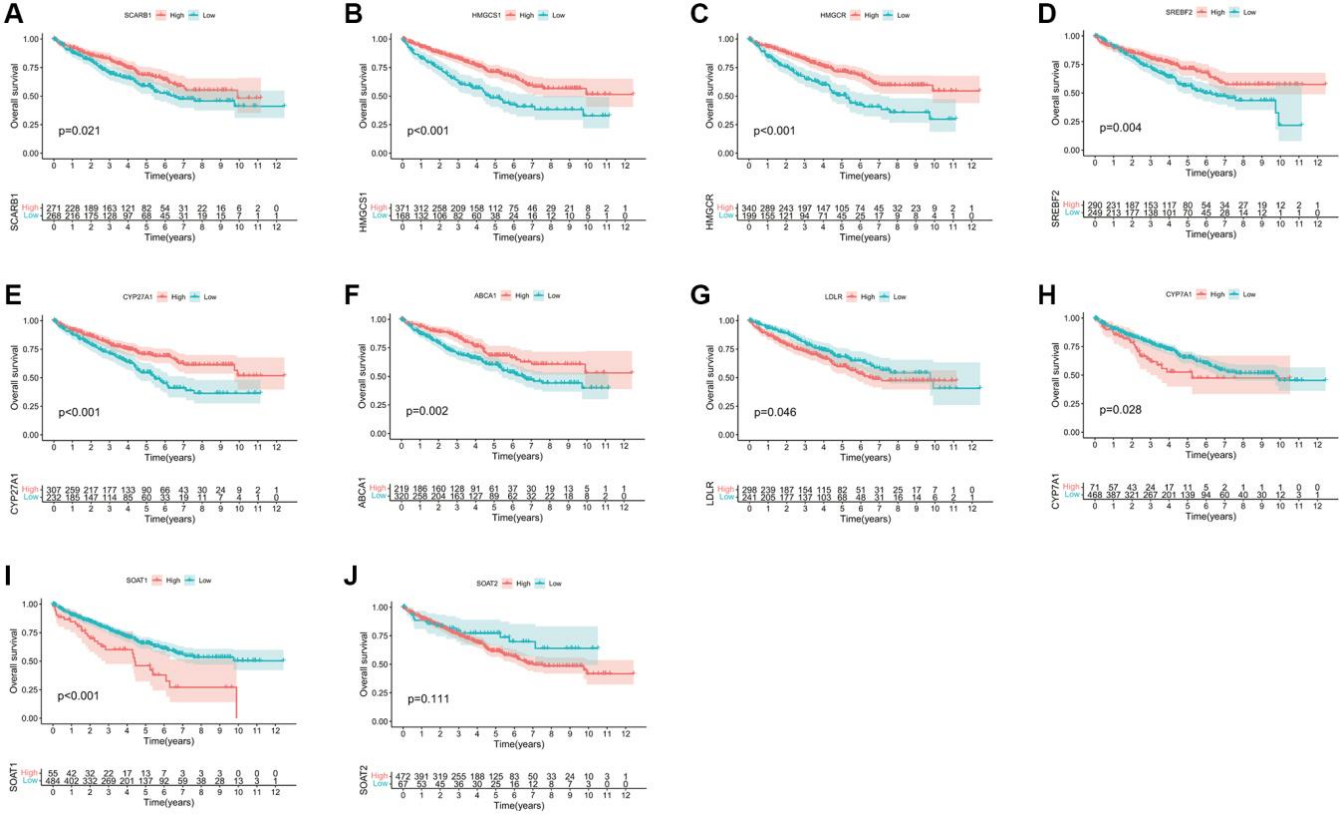
**Survival analyses for each CMG based on 539 patients with ccRCC from the TCGA database.** (**A**) SCARB1; (**B**) HMGCS1; (**C**) HMGCR; (**D**) SREBF2; (**E**) CYP27A1; (**F**) ABCA1; (**G**) LDLR; (**H**) CYP7A1; (**I**) SOAT1; (**J**) SOAT2. Kaplan-Meier curves with *p* < 0.05 showed a significant difference in survival probability to ccRCC patients.

**Figure 2:**
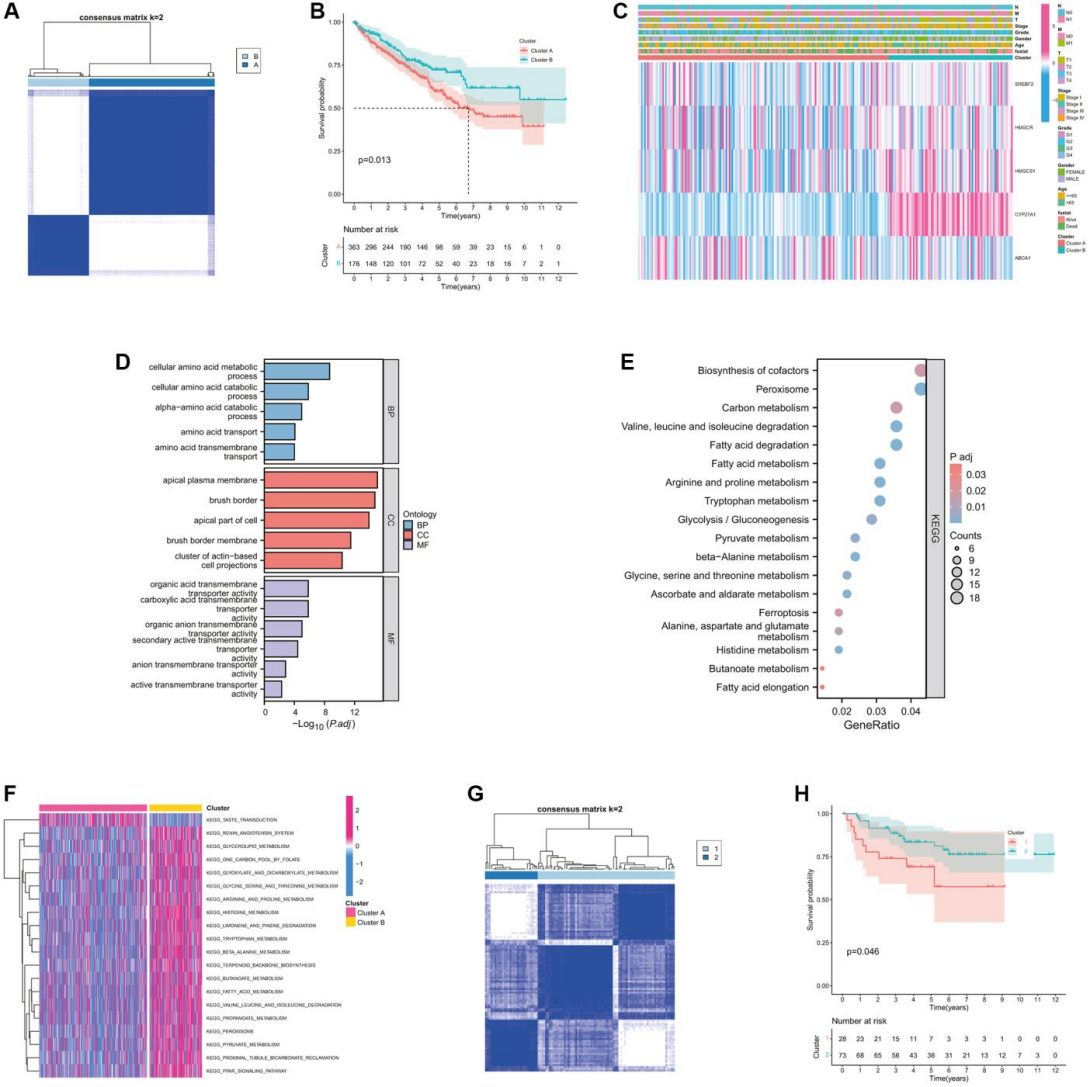
**Identification and comparison of various cholesterol metabolic subtypes of ccRCC.** (**A**) Consensus clustering matrix for *k* = 2. (**B**) The K-M survival curve showing the poorer survival probability of ccRCC patients in cluster A (*p* < 0.05). (**C**) The heatmap showing the clinicopathological characteristics, cluster situation, and expression levels of each survival-related CMGs of ccRCC samples. (**D**) GO functional annotation analysis of DEGs between clusters A and B, including enriched biological processes (BP), cellular components (CC), and molecular functions (MF). (**E**) KEGG pathway enrichment of DEGs between clusters A and B. The enriched items were analyzed by using gene counts, gene ratio, and adjusted *p* values. (**F**) The heatmap showing the biological pathways associated with distinct cholesterol metabolic modification subtypes by GSVA enrichment analysis. (**G**) Consensus clustering matrix for *k* = 2 in the E-MTAB-1980 cohort. (**H**) The K-M survival curve showing the poorer survival probability of ccRCC patients in cluster 1 from the E-MTAB-1980 cohort (*p* < 0.05).

The whole research design was illustrated in Supplementary Figure 1. Initially, we examined the associations between these CMGs and the outcome of ccRCC patients. The prognosis of ccRCC patients showed a significant correlation with the expression levels of 9 CMGs, as revealed by our analysis. Patients with elevated expression levels of SCARB1, HMGCS1, HMGCR, SREBF2, CYP27A1, and ABCA1 demonstrated improved overall survival (OS) (Figure 1A–1F). Conversely, patients displaying heightened expression of LDLR, CYP7A1, and SOAT1 exhibited poorer overall survival OS (Figure 1G–1J). Univariate Cox regression analysis was performed for these 9 genes, and finally 5 CMGs with stronger prognostic value were obtained. We conducted cluster analysis to examine the potential synergistic impact of these five survival-related CMGs on ccRCC. Accordingly, ccRCC patients from the TCGA cohort were sorted into clusters A (*n* = 467), and B (*n* = 72) under the clustering coefficient *k* = 2 (Figure 2A). At this point, the clusters were evidently differentiated from one another, exhibiting a strong internal cluster coherence. Further survival analysis indicated that patients belonging to cluster A experienced a more unfavorable prognosis (*p* < 0.05; Figure 2B). A heatmap depicted the expression patterns of the 5 CMGs and the corresponding clinical characteristics within each cluster (Figure 2C). To further investigate the heterogeneity of each lactate cluster, we identified 924 DEGs and then conducted functional enrichment analysis for these DEGs. In terms of biological processes (BPs) of GO analysis, the DEGs were principally involved in cellular amino acid metabolic and catabolic processes. In terms of cellular components (CCs), the DEGs were mainly enriched in the apical plasma membrane and brush border. In terms of molecular functions (MFs), the DEGs were significantly enriched in the organic acid and carboxylic acid transmembrane transporter activity (Figure 2D). At the same time, the KEGG pathway analysis showed the DEGs were enriched in biosynthesis of cofactors, peroxisome, and carbon metabolism (Figure 2E). GSVA analysis showed that cluster B was markedly enriched in pathways associated with activation of the nutrient metabolism, including glycerolipid metabolism, β-alanine metabolism, fatty acid, and propanoate metabolism (Figure 2F). To confirm the reliability of molecular classification, the E-MTAB-1980 cohort underwent the identical classification pipeline, and the KM analysis yielded consistent outcomes as the TCGA cohort (Figure 2G, 2H).

### Immune-related features of different cholesterol metabolic subtypes

**Figure 3:**
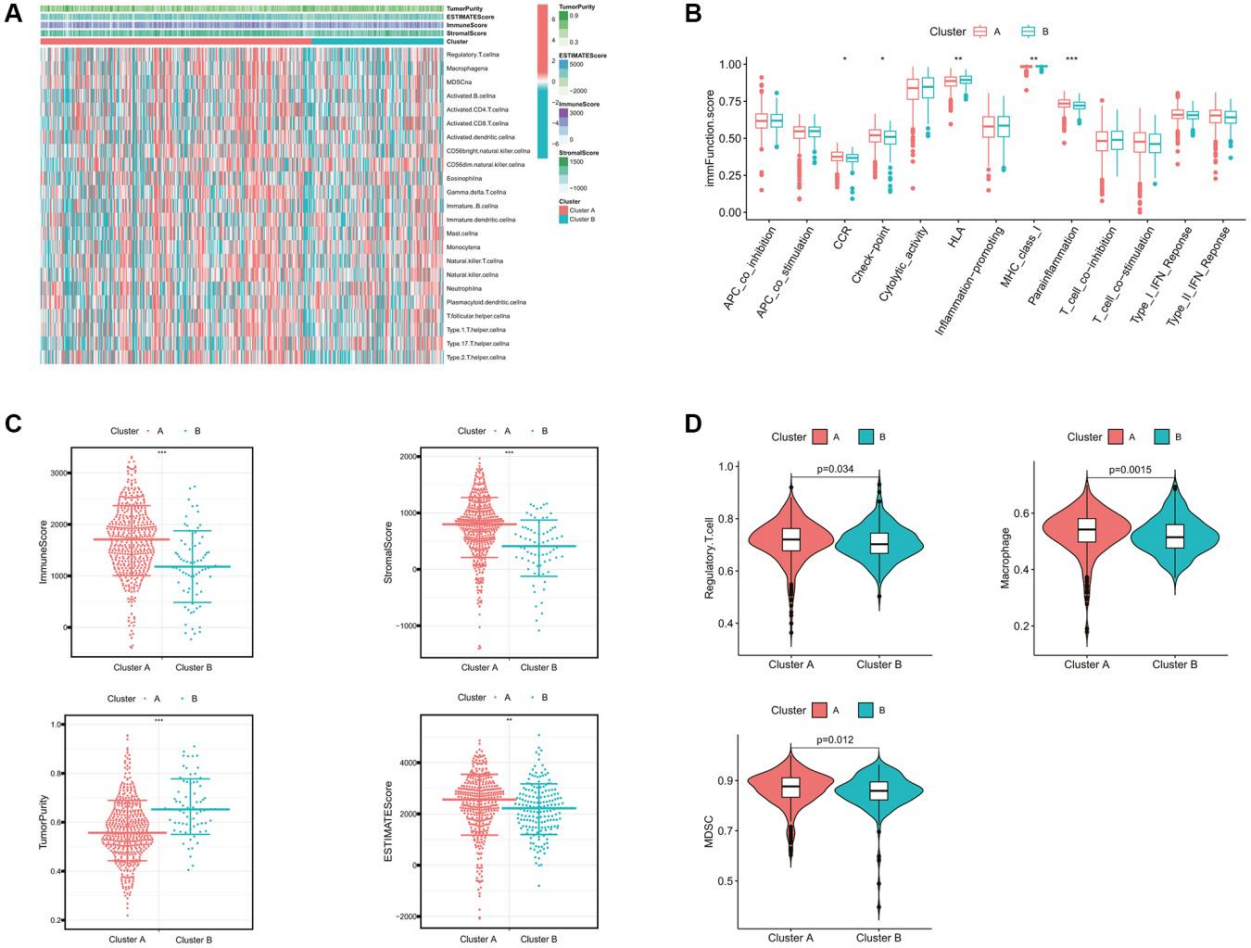
**Comparison of the immune characteristics between cholesterol metabolic subtypes.** (**A**) The heatmap showing the immune-related scores and immune cell infiltration levels of ccRCC samples. (**B**) Differential immune function analysis between cholesterol metabolic subtypes. (**C**) Differential analysis of immune-related scores between cholesterol metabolic subtypes based on the ESTIMATE algorithm. (**D**) Differential abundance analysis of major immunosuppressive cells between cholesterol metabolic subtypes based on the ssGSEA algorithm. ^*^p-value < 0.05, ^**^p-value < 0.01, ^***^p-value < 0.001.

Furthermore, we evaluated the correlation between the cholesterol metabolic clusters and the immune infiltration in the tumor microenvironment (TME). The heatmap in Figure 3A demonstrated the distribution of immune cell infiltration abundance and ESTIMATE-related scores between the two clusters for each ccRCC sample. In terms of immune function, the scores for CCR, check-point, and parainflammation were significantly higher in cluster A than in cluster B (Figure 3B). Interestingly, Figure 3C showed that patients in cluster A demonstrated a higher immune score, stromal score, and ESTIMATE score than those in cluster B. This seemed to be inconsistent with the poor prognosis of patients in cluster A. As mentioned previously, we noted that cluster A appeared to exhibit stronger immune checkpoint activity. Similarly, the main immunosuppressive infiltrating cells such as myeloid-derived suppressor cells (MDSCs), macrophages, and regulatory T cells in cluster A were significantly higher than those in cluster B (Figure 3D). These findings revealed that the cholesterol metabolic subtypes might be associated with the immune status of ccRCC patients.

### Construction and validation of the cholesterol metabolic signature

**Figure 4:**
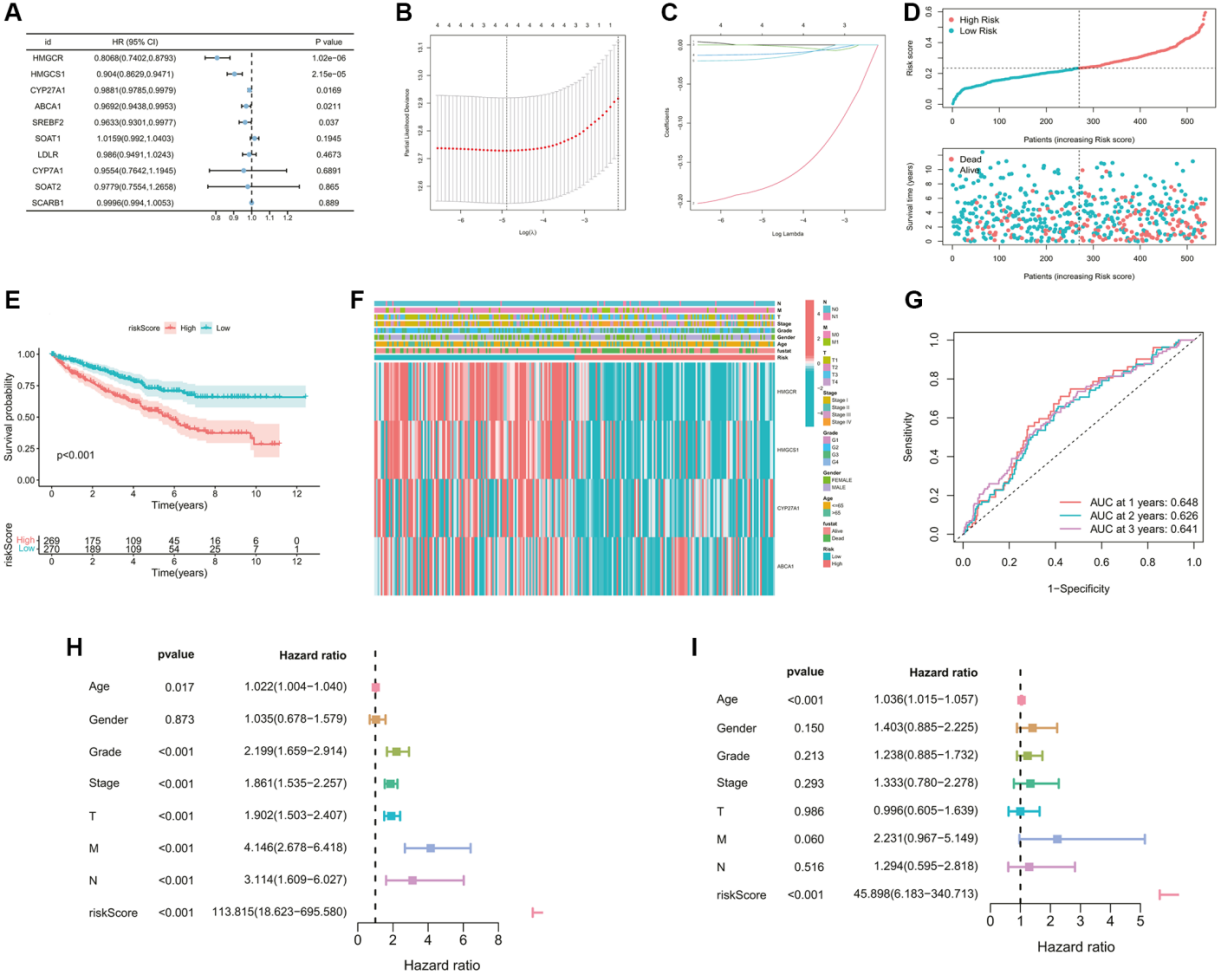
**Construction of the cholesterol metabolic prognostic signature.** (**A**) 5 prognostic genes were selected by the univariate Cox regression analysis (*p*-value < 0.05). (**B**, **C**) A 5-mRNA signature was constructed by the LASSO Cox regression. (**D**) The curve of risk score and survival status of the patients showing a positive correlation between mortality and risk score. (**E**) The K-M survival curve showing the poorer survival probability of ccRCC patients in the high-risk group (*p* < 0.001). (**F**) The heatmap showing the clinicopathological parameters, risk grouping situation, and expression levels of 4 model genes of ccRCC samples. (**G**) The AUC value of the ROC curve for predicting CM patients’ prognosis, indicating the robustness and accuracy of this 4-mRNA signature. (**H**, **I**) Predictive independence assessment of classical clinical predictors and our risk score by the (**H**) univariate and (**I**) multivariate Cox regression analysis.

The prognostic analysis was firstly carried out to filtrate CMGs significantly related to outcomes in ccRCC patients. As shown in the forest plot (Figure 4A), 5 CMGs were found to be associated with prognosis (*p* < 0.05). Then the LASSO regression analysis was applied in succession to single out the best model with the TCGA dataset (Figure 4B, 4C). The risk score for out signature was worked out through the established formula method:


$$\begin{array}{cc} Risk\;score & =(-0.18220)\times exp^{HMGCR}+(-0.00081)\\ & \times \;exp^{HMGCS1}+(-0.01731)\\ & \times \;exp^{\text{ABCA1}}+(-0.01171)\\ & \times \;exp^{\text{CYP27A1}} \end{array}$$

The risk score was then calculated for each case. Its mean threshold divided the ccRCC patients into two subgroups (low-risk (*n* = 270) and high-risk (*n* = 269)). Riskscore showed a negative correlation with patient survival based on the distribution of Riskscore in ccRCC samples (Figure 4D). KM analysis confirmed a lower likelihood of survival in the high-risk group (*p* < 0.05, Figure 4E). The heatmap visualized the differences in gene expression profile and clinical characteristics between the two risk subgroups (Figure 4F). An evaluation of the predictive performance was conducted with the time-dependent ROC curve of the model. The AUCs reached 0.648 in 1 year, 0.626 in 2 years, and 0.641 in 3 years (Figure 4G), which confirmed the stability of the predictive efficiency of our model. Univariate and multivariate Cox analyses were also performed to further explore the independent predictive ability of our signature. In the univariate Cox regression analysis, age, grade, overall stage, pathological T, N, M stage, and risk score were associated with the OS rates of ccRCC patients (all *p* < 0.05, Figure 4H). Furthermore, multivariate Cox analysis revealed that only age as well as the risk score of our signature (hazard ratio, 74.126; CI, 11.505 to 477.592; *p*-value < 0.001) remained an independent prognostic factor for ccRCC (Figure 4I).

### Immune-related features of the cholesterol metabolic signature

**Figure 5:**
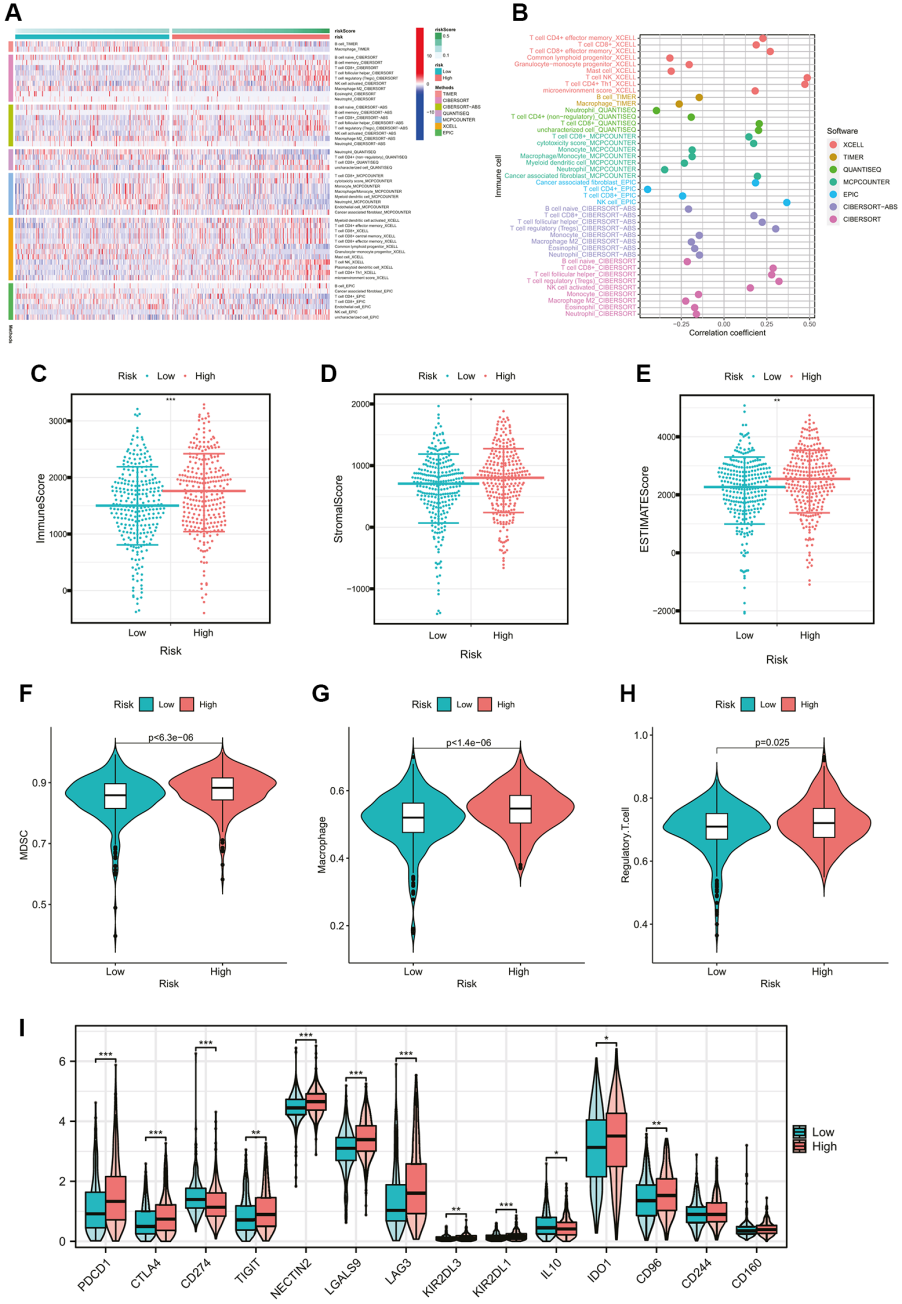
**Comparison of immune cells and immune functions of ccRCC patients between risk groups.** (**A**) The heatmap showing the tumor-infiltrating immune cells and risk scores by 7 mainstream algorithms. (**B**) The lollipop plot showing the correlation coefficients of tumor-infiltrating immune cells with CMGs-based risk scores. (**C**–**E**) Differential analysis of immune-related scores between risk groups based on the ESTIMATE algorithm. (**F**–**H**) Differential abundance analysis of major immunosuppressive infiltrating cells (MDSCs, macrophages, and Tregs) between risk groups based on the ssGSEA algorithm. (**I**) Differential expression analysis of the common immune checkpoint molecules between risk groups (PDCD1, *p* < 0.001; CTLA-4, *p* < 0.001; CD96, *p* < 0.001). ^*^p-value < 0.05, ^**^p-value < 0.01, ^***^p-value < 0.001.

In order to gain a deeper comprehension of the fundamental connection between the risk score and the immune composition of the ccRCC samples, a comparison was made between the low- and high-risk groups regarding the disparities in the different immune cell constituents. The Heatmap in Figure 5A computed by seven mainstream algorithms demonstrated the expression of immune cell infiltration abundance in each ccRCC sample. The correlation coefficients of the components with CMGs-based risk scores were calculated using Spearman’s analysis and visualized in a lollipop plot (Figure 5B). Subsequently, the immune, stromal, and ESTIMATE scores for the low- and high-risk groups were calculated to assess the overall immune status (Figure 5C–5E). The high-risk group exhibited an elevated immune score, stromal score, and ESTIMATE score, suggesting that numerous immune cells and molecules related to the immune system were plentiful in the high-risk group. Besides, the correlations of our risk score with the main immunosuppressive factors were also calculated. The results indicated that high-risk score was associated with higher-degree infiltration of immunosuppressive cells, such as MDSCs, Tregs, and macrophages (all *p* < 0.05, Figure 5F–5H), as well as higher expression of immune checkpoint molecules, such as PDCD1, CTLA4, CD96 and so on (all *p* < 0.05, Figure 5I). The findings unveiled the immune characteristic of our signature, indicating that individuals with elevated risk scores were more likely to undergo the transformation into the immunosuppressive tumor microenvironment (TME). This transformation was characterized by a significant increase in the presence of immunosuppressive cells and elevated levels of immune checkpoint molecules. This may provide positive guidance for immunotherapies targeting immune checkpoint molecules.

### Discovery and validation of exogenous cholesterol dependence in ccRCC

**Figure 6:**
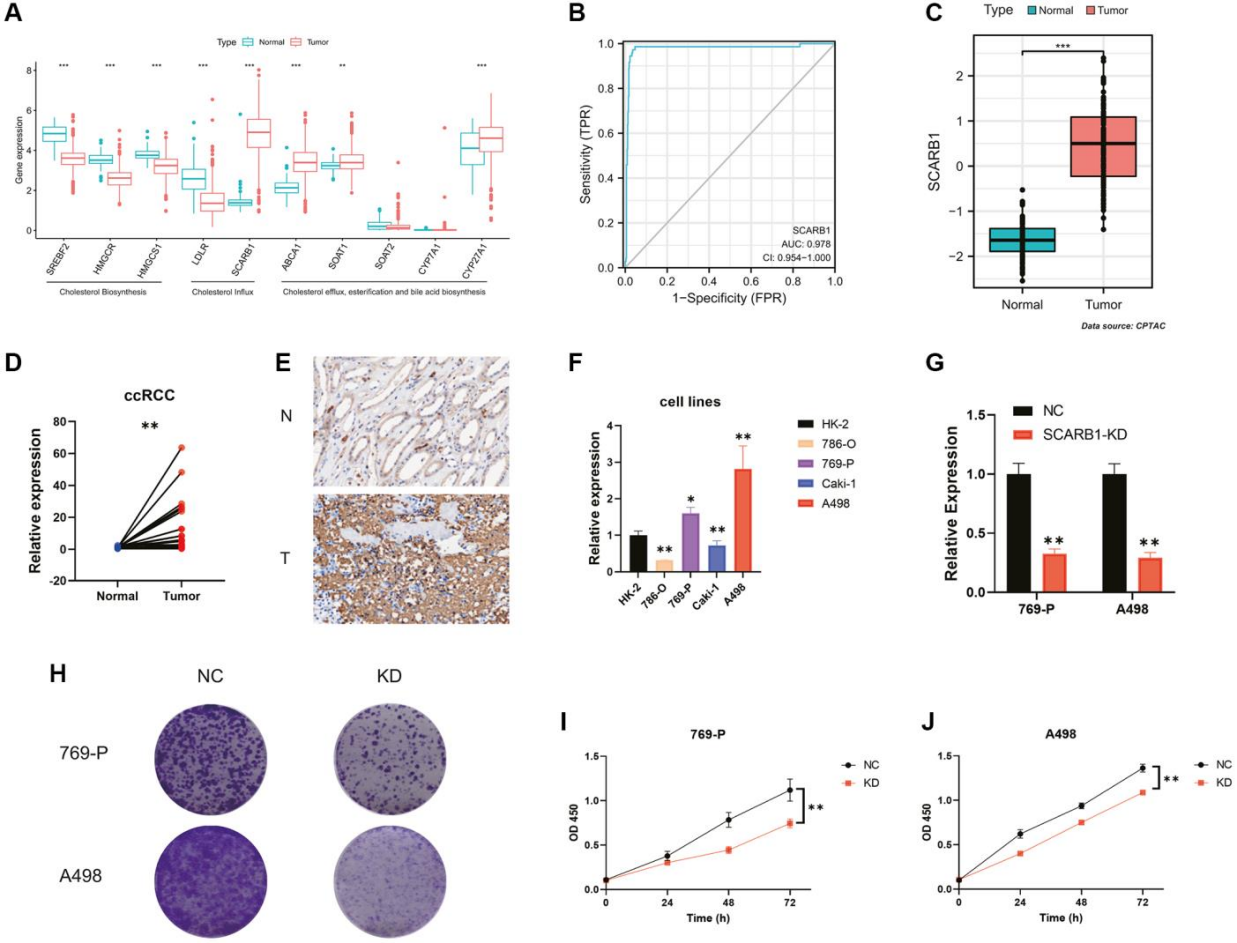
**Screening and validation of the effects of SCARB1 in ccRCC.** (**A**) Differential mRNA expression analysis of 10 CMGs between ccRCC and normal kidney tissues. (**B**) The AUC for SCARB1 in ccRCC cases was 0.9597. (**C**) Differential protein expression analysis of SCARB1 between ccRCC and adjacent cancerous tissues. (**D**) Comparison of mRNA expression levels of SCARB1 between ccRCC and adjacent cancerous tissues; *n* = 30. (**E**) IHC staining of SCARB1 in clinical ccRCC and adjacent cancerous tissues; N: adjacent cancerous tissue; T: ccRCC tissues. (**F**) Comparison of mRNA expression levels of SCARB1 in HK-2 and RCC cell lines by RT-qPCR; *n* = 3. (**G**) The knockdown efficiency of si-SCARB1 was verified by evaluating SCARB1 mRNA expression using RT-qPCR; *n* = 3. (**H**) Comparison of the clone formation ability between negative control groups and SCARB1 knockdown groups in 769-P and A498 cells. Abbreviations: NC: negative control; KD: knockdown; OD: optical density. (**I**, **J**) Cell proliferation curve following transfection of si-SCARB1 in (**I**) 769-P and (**J**) A498 cells. ^*^p-value < 0.05, ^**^p-value < 0.01, ^***^p-value < 0.001.

To define the characteristics of cholesterol metabolism in ccRCC, we investigated the mRNA expression of all 10 CMGs between normal and ccRCC samples. Based on their roles in cellular cholesterol metabolism, we classified the 10 CMGs into three categories based on the functions they performed: cholesterol biosynthesis (SREBF2, HMGCR, and HMGCS1) and cholesterol influx (LDLR and SCARB1) for cellular cholesterol sources, and cholesterol efflux, esterification, and bile acid biosynthesis (ABCA1, SOAT1, SOAT2, CYP7A1, and CYP27A1) for cellular cholesterol consumption. Compared to normal kidney tissues, SCARB1, ABCA1, SOAT1, and CYP27A1 demonstrated higher expression in ccRCC tissues (Figure 6A). In other words, only the SCARB1-mediated exogenous cholesterol uptake pathway was upregulated among the intracellular cholesterol source pathways. This phenomenon revealed that ccRCC may present an exogenous cholesterol-dependent characteristic and that the primary mediator of it, SCARB1, may have the potential to be the diagnostic marker and therapeutic target.

Figure 6B demonstrated a high diagnostic specificity with an AUC of 0.9597 for SCARB1 in ccRCC cases. The protein expression data from the CPTAC database further validated that SCARB1 exhibited significantly elevated expression at the translational level in ccRCC tissues, in comparison to the adjacent normal tissues (Figure 6C). Subsequently, RT-qPCR and IHC were performed. The results demonstrated that, compared with the adjacent normal tissues, the mRNA expression level of SCARB1 was significantly increased in ccRCC tissues (*p* < 0.01, Figure 6D). Through IHC, the protein expression of SCARB1 was detected on tumor cells, which was consistent with previous bioinformatics data (Figure 6E). Thus, we considered that SCARB1 may be a potential diagnostic biomarker for ccRCC and could be a promising therapeutic target.

Next, we examined SCARB1 mRNA levels in ccRCC cell lines. The results in Figure 6F showed that SCARB1 was highly expressed in 769-P and A498 cells, but lowly expressed in 786-O and Caki-1 cells. Thus, we used 769-P and A498 cell lines for loss-of-function assays. The si-SCARB1 molecule was employed to effectively downregulate SCARB1 mRNA expression in both cell lines (all *p* < 0.01, Figure 6G). The knockdown of SCARB1 significantly inhibited the clone formation in 769-P and A498 cells (Figure 6H). Additionally, the CCK-8 assays were performed, and the results revealed differences in cell proliferation at 24, 48, and 72 hours in each group. Compared with the cells in the negative control groups, the cell proliferation was significantly inhibited at all time points once 769-P and A498 cells were transfected with si-SCARB1 (all *p* < 0.01, Figure 6I, 6J).

### Exploration of the exogenous cholesterol dependence in SKCM

**Figure 7:**
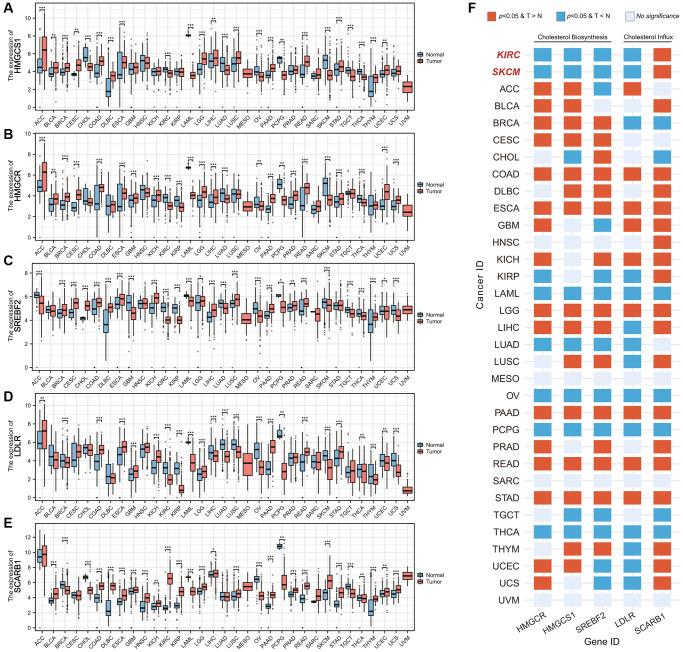
**Pancancer analysis of cholesterol-resource related gene expression.** (**A**–**E**) Differential mRNA expression analysis of (**A**) HMGCS1, (**B**) HMGCR, (**C**) SREBF2, (**D**) LDLR, and (**E**) SCARB1 between malignant tumor and corresponding adjacent tissues. (**F**) The heatmap showing the differences in the expression of each cholesterol-resource related gene in each malignant tumor and corresponding adjacent tissues. Abbreviations: T > N: The gene expression in the tumor is higher than the adjacent tissue; T < N: The gene expression in the tumor is lower than the adjacent tissue. ^*^p-value < 0.05, ^**^p-value < 0.01, ^***^p-value < 0.001.

The exogenous cholesterol-dependent characteristic of ccRCC aroused our interest, and we wondered whether this phenomenon was unique among common malignant tumors. Based on the data of the TCGA database, we analyzed the difference in expression levels of genes related to cholesterol sources in 33 common malignant tumors, including 3 genes mediating cholesterol biosynthesis and 2 genes mediating cholesterol intake (Figure 7A–7E). Figure 7F summarized the results of differential analysis of 5 genes. We found that besides ccRCC (KIRC for cancer id), skin cutaneous melanoma (SKCM) also exhibited the SCARB1-mediated exogenous cholesterol dependent characteristics.

**Figure 8:**
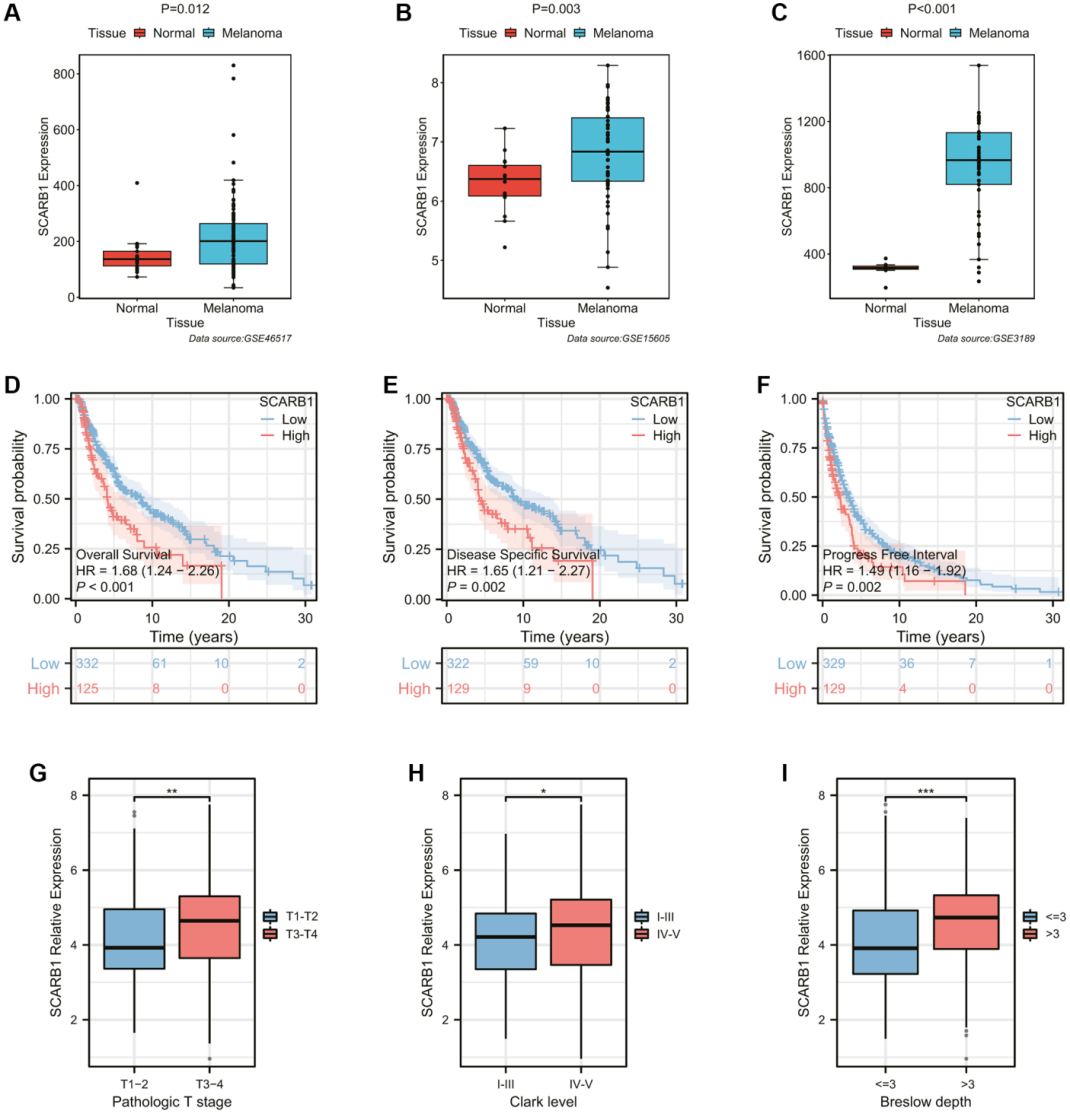
**Exploration of the expression and prognostic ability of SCARB1 in SKCM.** (**A**–**C**) Differential mRNA expression analysis of SCARB1 between cutaneous melanoma and normal skin tissues from the GEO database, including (**A**) GSE46517, (**B**) GSE15605, and (**C**) GSE3189. (**D**–**F**) The K-M survival curves showing the poorer survival probability of SKCM patients in the high-SCARB1 group with different observation end points, including (**D**) overall survival, (**E**) disease specific survival, and (**F**) progress free interval. (**G**–**I**) Differential mRNA expression analysis of SCARB1 between SKCM patients stratified by classic prognostic predictors, including (**G**) pathologic T stage, (**H**) Clark level, and (**I**) Breslow depth. ^*^p-value < 0.05, ^**^p-value < 0.01, ^***^p-value < 0.001.

Firstly, three SKCM cohorts from the GEO database were employed to verify the difference of SCARB1 expression between SKCM and normal skin tissues. In all cohorts, the expression of SCARB1 in SKCM was significantly higher than that in adjacent normal tissues (all *p* < 0.05, Figure 8A–8C). Then, the survival differences between SKCM patients in high- and low-SCARB1 expression groups were investigated with different survival indicators used as observation outcomes. SKCM patients with high SCARB1 expression possessed worse OS, disease specific survival (DSS), and progress free interval (PFI) in Figure 8D–8F (all *p* < 0.05). The differences in SCARB1 expression among SKCM patients stratified based on different classic prognostic factors were also explored. SKCM patients with advanced tumor state (pathologic T stage 3–4, Clark level IV-V, and Breslow depth >3 mm) tended to express more abundant SCARB1 significantly (all *p* < 0.05, Figure 8G–8I). The above results indicated that, consistent with ccRCC, the expression of SCARB1 was also an important prognostic factor in SKCM, playing a promoting role in the occurrence and development of SKCM.

In addition, we also investigated the relationship between the expression of SCARB1 and the abundance of immune cell infiltration in SKCM. We noticed that high-SCARB1 subgroup had poor immune cell infiltration, such as activated CD8 T cell, eosinophil, immature B cell, MDSC, macrophage and so on (Supplementary Figure 2A). After calculating the correlation coefficient between various immune cells and SCARB1 expression, only the abundance of CD58dim natural killer cells was positively correlated with SCARB1 expression, while the abundance of most other immune cells was negatively correlated with SCARB1 expression (Supplementary Figure 2B). By using the ESTIMATE algorithm to evaluate the overall immune status of TME, we found that patients in the high-SCARB1 subgroup often had lower immune scores, stromal scores, and ESTIMATE scores (all *p* < 0.05, Supplementary Figure 2C). And the expression of SCARB1 is significantly negatively correlated with the values of these three scores (all *r* < 0, Supplementary Figure 2D). Therefore, considering the poor prognosis of SKCM patients in high-SCARB1 subgroup, we hypothesized that SKCM patients in the high-SCARB1 subgroup exhibit a higher degree of immunosuppression, and SCARB1 may promote tumor occurrence and development in SKCM by affecting the recruitment and activation of immune cells. However, it is still a mystery whether this phenomenon is the subsequent influence of reprogramming of cholesterol metabolism or independent of abnormal lipid metabolism.

## DISCUSSION

To survive within the TME characterized by limited nutrient supply and metabolic waste accumulation, cancer cells adopt various metabolic adaptations to support their proliferation [23]. A common feature of metabolic reprogramming is the ability to obtain essential nutrients from nutrient deficient environments and utilize these nutrients to maintain survival and generate new biomass [24]. Among these adaptive methods, abnormal activation and reprogramming of cholesterol metabolism pathways are one of the hallmarks. As a rapidly proliferating cell, cancer cells need high levels of cholesterol to maintain cell membrane biogenesis and other functional requirement [18]. In the presence of lipid or oxygen limitations, the main transcription factor SREBP2 and its downstream targets are significantly upregulated in tumors, thereby enhancing cholesterol metabolism and supporting cancer progression. In this study, we systematically explored the cholesterol metabolism in ccRCC and SKCM and its impact on the TME.

First, we found that metabolic subtypes as well as risk score of the cholesterol metabolic signature had certain guiding significance for the prognosis of ccRCC and SKCM. A previous TCGA analysis of ccRCC highlighted the key role of metabolic alteration in ccRCC progression [25]. Many prognostic models have been developed based on important alterations in the metabolic processes of ccRCC. Focusing on the role of glycolysis in tumorigenesis, Xing et al. established a novel glycolysis-related gene signature that could predict OS for ccRCC [26]. A hypoxia-associated prognostic signature was developed by Li et al. in their study of the hypoxia and immune status of the TME in ccRCC [27]. In addition, an attempt was made by Wei et al. to assess the correlation between immune response and glutamine metabolic genes by developing a predictive model [28]. In the past few years, numerous research studies have provided insights into the alteration of cholesterol metabolism in cancer [29, 30], although this aspect has not been given equal focus in ccRCC compared to other types of cancers. The outcomes of our study led to an innovative avenue of research.

Moreover, CMGs showed differential expression in both ccRCC and SKCM, verifying the presence of metabolic reprogramming within the cholesterol metabolism in these two malignant tumors. These findings are comparable to the outcomes of prior studies. Wu G et al. reported that RCC possessed HMGCR and HMGCS as protective factors, distinguishing it from other cancers [31]. In a recent investigation concerning LDLR mutations decreasing the likelihood of ccRCC, it was also observed that the expression level of LDLR was suppressed in ccRCC cells [32]. In a single-cell sequencing study, Zhang et al. found that the melanoma cells exhibit obvious heterogeneity, including five functional subgroups related to cholesterol metabolism, Wnt signaling pathway, cell cycle, TGF beta signaling pathway and type I interferon [33]. In addition, cholesterol overload related to GRAMD1B in melanoma would activate AP-1 program to promote tumor invasion [34]. These results suggest the reprogramming of cholesterol metabolism in both ccRCC and SKCM, which could potentially contribute to the advancement of tumors.

Furthermore, we found that renal cancer cells and skin melanoma cells may have a greater tendency to acquire essential cholesterol via the uptake pathway. Qi et al. described the similar phenomenon in their study [35]. In ccRCC, there was a relatively higher expression of genes that controlled the production of fatty acids, such as FASN, SCD-1, and SREBP, while the expression of crucial genes that regulated the synthesis of cholesterol, like HMGCR and HMGCS, was reduced. It was speculated that the high abundance of cholesterol in ccRCC cells might be attributed to the uptake of cholesterol from external sources rather than its synthesis within the cells. In the meantime, it was discovered that LXR owned the ability to increase the expression of SCARB1, which was accountable for the absorption of HDL, while simultaneously decreasing the expression of LDLR, which was responsible for the absorption of LDL. There was also speculation that ccRCC could maintain the cholesterol levels inside cells by consuming a significant quantity of HDL.

Finally, our results suggested that abnormal cholesterol metabolism might affect the immune microenvironment of tumors, thereby interfering with tumor progression and immune response. Previous studies suggested that cholesterol metabolism was immune-related and reprogramming of cholesterol metabolism in cancer cells or Tumor-infiltrating immune cells (TIICs) in TME might have an influence on tumor immune recognition or immune escape [29, 36]. Within the TME, TIICs exhibit consistent and adaptable functions, influencing the behavior of cancer cells and displaying both anti-tumor and pro-tumor capabilities [37]. Several studies indicated that the elimination of cholesterol resulted in a notable rise in tumor growth, deterioration of mouse survival, reduction in the quantity of CD8 cells within the TME, and insignificant alteration in CD4 cell levels [38]. In our current research, we have found high risk scores of the cholesterol metabolic signature were associated with high-degree infiltration of immunosuppressive cells, such as MDSCs, Tregs, and macrophages. MDSCs are defined as bone marrow-derived populations of consistently immature cells that have the ability to significantly suppress T-cell responses and are the primary effector cells for tumor immunosuppression [39]. A mechanistic study has shown that 27-HC promoted the differentiation of MDSCs and increased the intratumoral abundance of MDSCs, thereby promoting tumor progression [40]. In addition to affecting differentiation, TME-derived chemokines can recruit MDSCs to infiltrate into the tumor microenvironment, such as CCL2, CCL5, and other chemokines secreted by breast, gastric, and ovarian cancers [39]. Macrophages are broadly classified as classically activated (M1) or alternative activated (M2) types with anti-tumor or pro-tumor properties. Tumor-associated macrophages (TAMs) often exhibit an M2-like phenotype with high expression of immunosuppressive molecules such as IL-10 [41, 42]. A study by Goossens et al. reported that tumor-derived hyaluronic acid upregulated ABCA1 in TAMs, leading to increased membrane cholesterol efflux [43]. Cell membrane cholesterol depletion and lipid raft structural disruption enhanced IL-4 signaling while IFN-γ signaling was impaired, thereby promoting the polarization of TAM toward an immunosuppressive M2-like phenotype. T lymphocytes play a central role in anti-tumor immunity. Cholesterol and its derivatives are important regulators of T lymphocyte function. A recent study found that high cholesterol secreted from tumor cells in the TME could also upregulate tumor-infiltrating CD8^+^ T cell cytoplasmic cholesterol content [44]. High cholesterol in CD8^+^ T cells subsequently triggered endoplasmic reticulum stress and upregulated the expression of endoplasmic reticulum stress-associated protein XBP1. And XBP1, as a transcription factor, promoted the transcription of immunosuppressive molecules such as PD-1, TIM-3, and LAG-3 in CD8^+^ T cells, thereby causing CD8^+^ T cells to exhibit a functionally depleted and immunosuppressive state and promoting tumor progression. To summarize, it is reasonable to speculate that there are undeniable connections among the metabolism of cholesterol, the immunity against tumors, and ccRCC. Nevertheless, the process of their collaboration requires additional investigation.

The strength of our study resided in our statistical analysis of the cholesterol metabolism-related genetic prognostic signature using high-throughput data and a large-scale database, which met the urgent need for a validation index for ccRCC. In addition, our study contributed to a better understanding of the role of cholesterol metabolism in ccRCC and SKCM. Inevitably, our study also has some limitations. First, clinical parameters such as age, and pathological stage were not included in our risk score formula. Second, the clinical information in the TCGA database was not comprehensive and we could not obtain additional parameters to validate our model, such as CT images and renal function scores. Third, the mechanisms by which CMGs like SCARB1 affected the carcinogenesis and progression of ccRCC required further studies *in vivo* and *in vitro*. The main pathological feature of ccRCC is the accumulation of lipids in tumor cells, and the reasons for this accumulation and the molecular mechanisms that promote the occurrence and development of ccRCC are still unknown. In further research, we need to focus on whether the high expression of SCARB1 affects the lipid droplet accumulation in ccRCC. In addition, further research is needed to investigate the interference of lipid metabolism changes on the immune microenvironment.

## CONCLUSION

In conclusion, the cholesterol metabolic status of tumor cells in ccRCC patients was closely related to prognosis. We constructed and validated a cholesterol metabolism-related gene signature consisting of four genes to predict the prognosis of ccRCC, which was associated with immune infiltration. Both ccRCC and SKCM exhibit exogenous cholesterol dependent metabolic fragility, and targeting SCARB1, which mediates their main source of cholesterol, may aid in their diagnosis and accurate treatment.

## Supplementary Materials

Supplementary Figures
