# Supplementary material for: Bridging the gap between clear cell renal cell carcinoma and cutaneous melanoma: the role of SCARB1 in dysregulated cholesterol metabolism
Source: Aging (Albany NY). 2023 Oct 5;15(19):10370–88. doi: 10.18632/aging.205083 (PMC10599744; doi:10.18632/aging.205083)
Supplement: Supplementary Figures [file aging-15-205083-s001.pdf]

SUPPLEMENTARY FIGURES

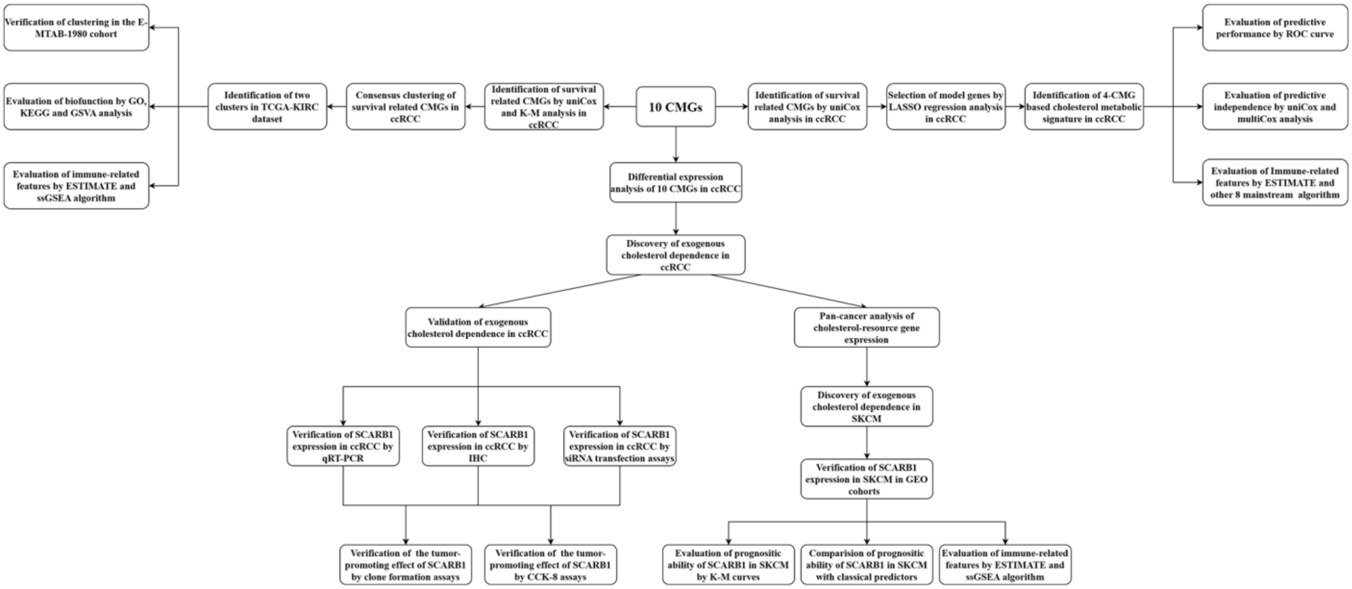

Supplementary Figure 1. The workflow of the present study.

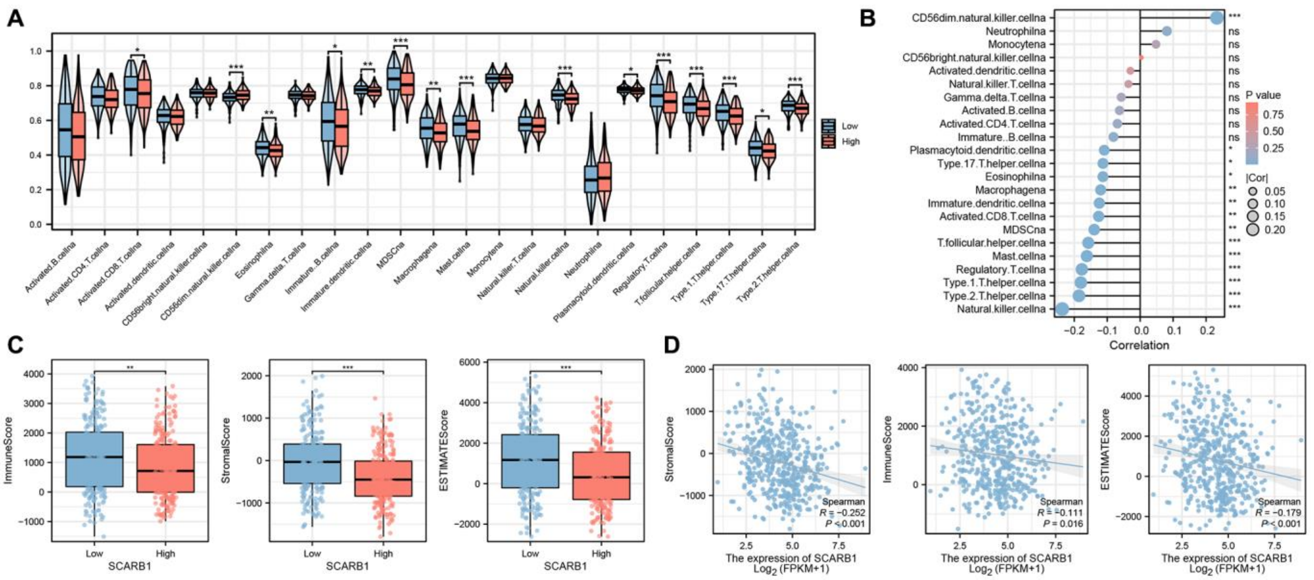

Supplementary Figure 2. Comparison of the immune characteristics between high- and low-SCARB1 groups in SKCM. (**A**) Differential abundance analysis of tumor-infiltrating immune cells between high- and low-SCARB1 groups in SKCM based on the ssGSEA algorithm. (**B**) Spearman correlation analysis of the tumor-infiltrating immune cells under SCARB1 expression in SKCM. (**C**) Differential analysis of immune-related scores between high- and low-SCARB1 groups in SKCM based on the ESTIMATE algorithm. (**D**) Spearman correlation analysis of immune-related scores under SCARB1 expression in SKCM.
